# Impact of Eccentric Residual Mitral Regurgitation on Long-Term Survival After Transcatheter Edge-to-Edge Mitral Valve Repair

**DOI:** 10.1016/j.shj.2025.100752

**Published:** 2025-10-30

**Authors:** Knut Qvale Søndenaa, Ola Alexander Schei, Rasmus Bach Sindre, Christian Engelsen Berg-Hansen, Barbara Rogge, Cecilie Linn Aas, Stig Urheim, Jon Herstad, Erik Packer, Vegard Tuseth, Dana Cramariuc

**Affiliations:** a Department of Clinical Science, University of Bergen, Bergen, Norway; b Department of Heart Disease, Haukeland University Hospital, Bergen, Norway; c The Cardiology Clinic Hjertelegen, Bergen, Norway; d Department of Anaesthesia and Intensive Care, Haukeland University Hospital, Bergen, Norway

**Keywords:** Eccentric regurgitation, Echocardiography, Edge-to-edge valve repair, Mitral regurgitation, Mitral valve repair

## Abstract

**Background:**

More than mild residual mitral regurgitation (MR) is associated with reduced survival after transcatheter edge-to-edge mitral valve repair (M-TEER). However, whether the morphology of the residual MR (rMR) jet affects long-term clinical outcomes has not been reported. The purpose of the study was to assess the impact of eccentric rMR (erMR) at discharge on long-term mortality and heart failure hospitalizations after M-TEER.

**Methods:**

We analyzed preprocedural, intraprocedural, and early postprocedural echocardiographic data (1-2 days post-TEER) in patients treated by M-TEER at our institution between 2012 and 2022. Eccentric rMR was defined as a nonsymmetric, wall-hugging MR jet.

**Results:**

Among 125 patients with early residual jets, erMR was present in 30% and more commonly in those with medially placed devices and in severe rMR (*p* < 0.05). Eccentric jets were equally frequent in patients with originally functional or degenerative MR. During 49 (26-79) months follow-up, the median survival was 29 months in patients with erMR vs. 65 months in patients without erMR (*p* < 0.001). In Cox regression analysis, erMR predicted a 2.2-fold higher risk of death (95% CI 1.3-3.7, *p* < 0.05) after adjustment for MR severity, age, mean blood pressure, atrial fibrillation, left ventricular and left atrial size, and device position. Eccentric rMR also predicted a 1.8-fold (95% CI 1.1-3.1, *p* = 0.02) higher adjusted risk of heart failure hospitalization post-M-TEER.

**Conclusions:**

Presence of erMR at discharge is associated with reduced long-term survival after M-TEER independent of the severity of rMR. Our findings shed light on the importance of preprocedural and intraprocedural planning to avoid eccentric jets and mitigate unfavorable long-term outcomes after M-TEER.

## Highlights

• Eccentric mitral regurgitation affects 30% of patients after edge-to-edge repair.
• An eccentric jet is more common with medially placed devices and large jets.
• Eccentric regurgitation at discharge doubles the long-term risk of death.
• The negative prognostic impact is independent of residual regurgitation severity.
• Both the quantity and quality of residual jets need evaluation after valve repair.

## Introduction

Mitral regurgitation (MR) is the most common valvular heart disease globally and its prevalence continues to rise due to population aging.^1^ Untreated isolated MR is associated with high rates of adverse clinical outcomes including excess mortality.^2^ However, less than 25% of patients with significant MR benefit from surgical treatment during their lifetime, even when there is no echocardiographic evidence of left ventricular (LV) dysfunction.^2^

Transcatheter mitral valve repair with edge-to-edge leaflet approximation (transcatheter edge-to-edge mitral valve repair [M-TEER]) has established itself as a viable option in patients with severe symptomatic MR in whom surgery is prohibitive or the surgical risk is deemed to be high. Data suggest that recurrent MR is more frequent after M-TEER than after conventional surgical mitral valve repair or isolated surgical edge-to-edge repair (also known as Alfieri stitch).^3^^,^^4^ However, ongoing trials are currently testing whether M-TEER might be a treatment alternative for patients at a lower surgical risk.^5^ In the context of possible extending indications for M-TEER, it is increasingly important to understand how we can refine the preprocedural planning and improve its early results to leverage both its long-term efficacy and favorable patient outcomes.

In a large retrospective registry of patients with heart failure and secondary MR, presence of more than mild residual MR (rMR) after M-TEER was linked to worse 2-year survival.^6^ Patients with more than mild rMR have also been reported to have a higher risk of developing significant MR within the first 12 months after M-TEER and experience more frequent heart failure hospitalizations.^7^ In untreated MR, there is evidence that eccentric or “wall-hugging” jets are more challenging to quantify than the central ones and are associated with poorer outcomes.^8^ Whether the direction of an early rMR after M-TEER has an independent effect on the long-term survival following M-TEER has, to our knowledge, not been previously investigated.

The current study aimed to analyze the association between the presence of an eccentric rMR (erMR) at discharge and the long-term risk of death in a cohort of adults undergoing M-TEER at our institution and included in the Percutaneous Mitral Valve Repair in Norway study. Secondly, we investigated whether an erMR was an independent predictor of heart failure hospitalization after M-TEER. Moreover, we sought to identify the clinical, echocardiographical, and procedural factors specifically associated with the risk of developing erMR early after percutaneous mitral valve repair.

## Methods

### Study Population

The study population consisted of patients with significant (more than moderate) MR treated by M-TEER at our institution between 2012 and 2022. Of these, we selected only the cases that underwent a complete preoperative echocardiography at our Heart Valve Center within 3 months before M-TEER, underwent successful M-TEER using edge-to-edge repair devices from a single vendor, had high-quality intraoperative transesophageal acquisitions, and a complete echocardiography at discharge demonstrating trace or greater MR. Two patients required emergent surgical bailout due to intraprocedural complications and were excluded from the present analysis. Both patients with degenerative and functional MR were included. The presence and severity of rMR could be assessed in 125 of the 129 patients that fulfilled the inclusion criteria.

Data on patient symptoms, comorbidities, and daily medication was collected through chart review. History of atrial fibrillation included paroxysmal or chronic atrial fibrillation documented in the patients' electronic health records. The preoperative renal function was assessed by the estimated glomerular filtration rate calculated from the serum creatinine level, age, and sex using the Chronic Kidney Disease Epidemiology Collaboration equation. The preoperative serum level of the N-terminal pro-B-type natriuretic peptide (NT-proBNP) was an indicator of the severity of heart failure before M-TEER.

The study was approved by the regional ethics committee (2019/35092) and was conducted in accordance with the revised Declaration of Helsinki. The need for informed consent was waived due to the retrospective nature of the investigation. The data that support the findings of this study are available from the corresponding author on reasonable request.

### Echocardiographic Measurements

Standard transthoracic echocardiograms were performed before the intervention and at discharge by 2 experienced interventional echocardiologists using the same high-end ultrasound equipment (Vivid E90; GE Vingmed Ultrasound, Horten, Norway).^9^ All M-TEERs were guided by transesophageal echocardiography (TEE) performed by the same investigators. Both transthoracic and TEE acquisitions were analyzed on Echopac workstations (version 204; GE Vingmed Ultrasound, Horten, Norway).

**Figure 1:**
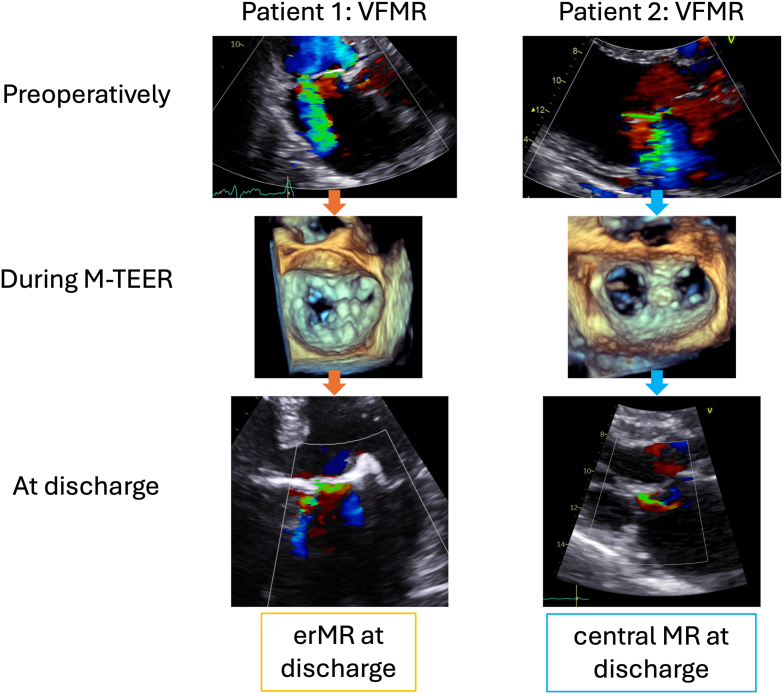
Preprocedural, intraprocedural, and postprocedural echocardiographic acquisitions in (Case 1) a patient with mainly central ventricular functional MR (VFMR) before M-TEER, treated with one central and one medial M-TEER device and presenting erMR at discharge. This patient died 2 years after M-TEER and (Case 2) a patient with eccentric VFMR before M-TEER, treated with one central M-TEER device and presenting mild central MR at discharge. This patient did not experience adverse clinical outcomes during follow-up. Abbreviations: erMR, eccentric mitral regurgitation; MR, mitral regurgitation; M-TEER, transcatheter edge-to-edge mitral valve repair.

MR severity was graded using a standardized, integrative method combining qualitative, semiquantitative and quantitative criteria (based on volumetric measurements) and performed by cardiologists with expertise in valvular heart disease diagnostics.^10^^,^^11^ The severity and eccentricity of the rMR jet (defined as a noncentral, nonsymmetric, wall-hugging jet) was ascertained at the discharge echocardiography (Figure 1).^11^ In challenging cases with multiple TEER devices and extensive intra-atrial acoustic shadowing, the eccentricity of rMR was also checked on the intraprocedural TEE acquisitions. The mean mitral gradient was measured intraoperatively and postoperatively.

The LV chamber size was measured at end-diastole and end-systole using both diameters and volumes.^12^ The LV systolic function was assessed by the Simpson's biplane ejection fraction (EF, low if <52% in men and <54% in women), and the LV pump performance by the Doppler stroke volume.^12^ The maximum left atrial (LA) diameter was measured at end-systole in the parasternal long-axis view.^13^

### M-TEER Procedural Features

The success of each leaflet grasping and the position of each mitral device were reviewed on the intraprocedural TEE acquisitions. Only patients with an adequate grasp of both leaflets (no single leaflet device attachment or loss of leaflet insertion detected) were selected for the present analysis.^7^ The M-TEER devices were classified as lateral if placed at the mitral segments A1/P1, central if at A2/P2, and medial if at A3/P3.

### Outcomes

We examined primarily the association between erMR at discharge and all-cause mortality. We defined hospitalizations for heart failure post-M-TEER as the secondary outcome. Patients were followed from the day of the M-TEER and until March 2025. All deaths and hospitalizations for heart failure were ascertained from medical records.

### Statistical Analyses

Statistical analyses were performed using the IBM SPSS Statistics 28.0 package (IBM Corp., Armonk, NY). Patients were grouped according to the presence or absence of erMR at discharge.

Normality of data distribution was evaluated using the Shapiro–Wilk test and by Q-Q plots. Comparisons between groups were performed by chi-square tests for categorical variables, independent-samples t-tests for normally distributed continuous variables, and Mann–Whitney U tests for continuous variables without a normal distribution. Results are reported as mean ± SD, median (25th-75th percentile), or percentages, as appropriate. Correlations were assessed by the Spearman test.

The relation between preoperative and intraoperative features and presence of erMR at discharge was first investigated in univariable regression analyses. Variables that were both clinically relevant and associated with erMR at *p* < 0.01 were then entered into a multivariable logistic regression analysis run with a backward stepwise procedure. The impact of erMR on the primary and secondary outcome was tested in Kaplan–Meier survival analyses with log-rank test for the overall analysis, as well as in univariable and multivariable Cox regression analyses. Results are reported as hazard ratios with 95% confidence intervals (CI). A two-tailed *p* < 0.05 was considered significant both in univariable and multivariable analyses.

## Results

### Clinical Characteristics

At the preoperative examination, the study patients were on average 77 years old, more commonly men, and highly symptomatic with 66% of them reporting dyspnea in New York Heart Association class III or IV. The serum level of NT-proBNP was also significantly increased (Table 1). More than half of the population had a history of revascularized coronary artery disease, 65% had known atrial fibrillation, and the majority was using daily heart failure medication (Table 1).

**Table 1:** Preoperative clinical characteristics of the whole population and of patients with vs. without early erMR after M-TEER

	Whole population (n = 125)	erMR+ (n = 38)	erMR- (n = 87)	*p*value
Age, years	77 [70-81]	79 [71-82]	77 [70-80]	0.24
Women	31%	26%	33%	0.44
Heart rate, bpm	74 ± 12	74 ± 12	73 ± 13	0.72
Systolic BP, mmHg	121 [105-136]	120 [104-130]	123 [107-139]	0.25
Diastolic BP, mmHg	67 [60-79]	64 [60-71]	70 [60-80]	**0.03**
Mean BP, mmHg	85 [76-95]	83 [75-89]	89 [78-100]	**0.05**
NYHA class III or IV	66%	68%	66%	0.46
NT-proBNP, pg/mL	2343 [1182-4440]	2575 [1279-5981]	2102 [1144-4243]	0.54
eGFR, ml/min/1,73 m^2^	51 [37-60]	50 [36-60]	51 [37-60]	0.95
History of hypertension	35%	37%	35%	0.80
Atrial fibrillation	65%	68%	63%	0.56
Diabetes mellitus	18%	18%	18%	0.99
Chronic obstructive pulmonary disease	14%	18%	12%	0.30
Coronary artery disease	59%	61%	59%	0.84
Medication				
Beta-blockers	90%	95%	89%	0.28
ACE inhibitor or ARB	82%	82%	83%	0.87
MRA	35%	26%	39%	0.17
Diuretic	86%	82%	89%	0.30

*Notes.* Values are median [25th-75th percentile], mean ± SD or percentages. *p* values represent the level of significance when comparing patients with vs. without erMR by Mann–Whitney U tests (for continuous variables without a normal distribution), independent-samples t-tests (for normally distributed continuous variables) or chi-square tests (for categorical variables). *p* values below 0.05 are marked in bold text.

Abbreviations: ACE, angiotensin-converting enzyme; ARB, angiotensin receptor blocker; BP, blood pressure; eGFR, estimated glomerular filtration rate; erMR, eccentric residual mitral regurgitation; MRA, mineralocorticoid receptor antagonist; M-TEER, transcatheter edge-to-edge mitral valve repair; NT-proBNP, N-terminal pro-B-type natriuretic peptide; NYHA, New York Heart Association functional class.

In this cohort, 30% presented erMR at discharge after M-TEER. Of these, 53% had eccentric MR preoperatively and 47% experienced new-onset erMR induced by M-TEER. Compared to patients with only trace MR (8%) or greater central rMR (62%), those with erMR had lower preoperative diastolic and mean blood pressure (BP). Their clinical profiles were otherwise comparable with no differences in age, heart rate, estimated glomerular filtration rate, or NT-proBNP, and with similar prevalence of relevant comorbidities and daily cardiovascular medication (Table 1).

### Echocardiographic and M-TEER-Related Characteristics

**Figure 2:**
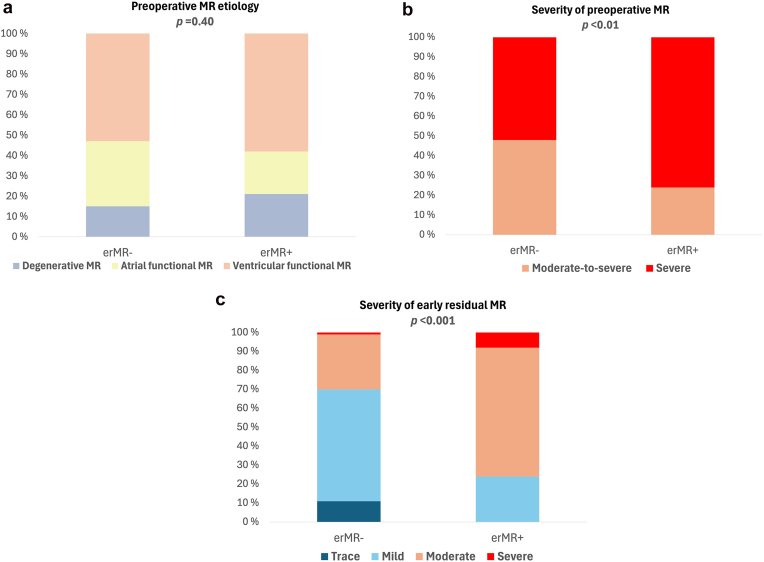
Comparison between patients with and without erMR at discharge after M-TEER with respect to (a) preoperative MR etiology; (b) preoperative MR severity; and (c) MR severity at discharge. *p* value for comparison between groups by chi-square test. Abbreviations: erMR, eccentric mitral regurgitation; MR, mitral regurgitation; M-TEER, transcatheter edge-to-edge mitral valve repair.

Patients with erMR at discharge had similar LA and LV size before the mitral valve intervention as patients without erMR and equally impaired EF and forward stroke volume (Table 2). The etiology of the preoperative MR did not differ between the groups, being mostly functional and in over half of the cases secondary to LV failure (Figure 2a). However, patients with erMR at discharge had more severe MR before M-TEER (*p* < 0.01, Figure 2b).

**Table 2:** Echocardiographic and procedural characteristics of the whole population and of patients with vs. without early erMR after M-TEER

	Whole population (n = 125)	erMR+ (n = 38)	erMR- (n = 87)	*p value*
Preoperative characteristics				
LV end-diastolic diameter (cm)	6.10 [5.40-6.70]	6.20 [5.48-6.85]	6.00 [5.20-6.60]	0.52
LV end-systolic diameter (cm)	4.61 [3.90-6.03]	4.90 [3.85-5.95]	4.60 [3.90-6.10]	0.99
LV end-diastolic volume, ml	179 [131-240]	178 [132-248]	185 [125-239]	0.75
LV end-systolic volume, ml	105 [60-164]	104 [60-180]	105 [59-163]	0.83
LV stroke volume/BSA, ml/m^2^	28 ± 8	28 ± 10	27 ± 8	0.75
LV EF, %	43 [28-55]	40 [27-58]	43 [30-53]	0.95
LA maximum diameter, cm	5.00 [4.60-5.40]	5.00 [4.80-5.30]	5.00 [4.60-5.50]	0.93
Tricuspid maximum pressure gradient, mmHg	36 [28-45]	36 [28-46]	35 [28-45]	0.50
Intraoperative characteristics				
Number of M-TEER devices				0.59
1 device	32%	26%	34%	
2 devices	60%	63%	59%	
3 or 4 devices	8%	11%	7%	
Position of M-TEER devices				
Lateral devices	7%	5%	8%	0.58
Central devices	90%	84%	93%	0.12
Only central	*76%*	*58%*	*85%*	
Combined central-lateral	*5%*	*3%*	*6%*	
Combined central-medial	*9%*	*23%*	*2%*	
Medial devices	13%	32%	5%	**<0.001**
Mean mitral gradient (mmHg)	3.0 [2.0-4.0]	3.0 [2.0-4.0]	3.0 [2.0-4.0]	0.61
Characteristics at discharge				
Mean mitral gradient (mmHg)	3.3 [2.1-4.6]	3.7 [2.7-6.0]	3.0 [2.0-4.0]	0.11
Severe rMR	3%	8%	1%	**<0.05**

*Notes.* Values are median [25th-75th percentile], mean ± SD, or percentages. *p* values represent the level of significance when comparing patients with vs. without erMR by Mann–Whitney U tests (for continuous variables without a normal distribution), independent-samples t-tests (for normally distributed continuous variables), or chi-square tests (for categorical variables). *p* values below 0.05 are marked in bold text.

Abbreviations: BSA, body surface area; EF, ejection fraction; erMR, eccentric residual mitral regurgitation; LA, left atrial; LV, left ventricular; M-TEER, transcatheter mitral edge-to-edge repair; rMR, residual mitral regurgitation.

During the intervention, 60% of the patients received 2 M-TEER devices, and roughly ¾ were treated entirely with central devices. The clip positioning did not differ between patients with originally degenerative, ventricular functional, or atrial functional MR. The total number of MitraClips was similar between the groups with vs. without early erMR. Even if less used overall, a medial device, alone or in combination with a central clip, was significantly more often employed in patients that subsequently presented erMR (*p* < 0.001, Table 2).

In univariable logistic regression analyses, the preoperative and intraoperative characteristics related to erMR at discharge were lower diastolic BP, implantation of a medial device, and more severe preoperative MR (all *p* < 0.05). When analyzed concomitantly in a multivariable logistic regression analysis with stepwise removal of nonsignificant covariables, only severe preoperative MR (odds ratio 2.8 [95% CI 1.1-6.9], *p* = 0.03) and placement of a medial clip (odds ratio 8.9 [95% CI 2.6-30.9], *p* < 0.001) retained a significant association with erMR (Nagelkerke R^2^ 0.22, *p* < 0.001 for the overall model).

Postoperatively, patients with erMR at discharge had more often moderate or severe MR compared to those without erMR (*p* < 0.001, Figure 2c).

### Impact of erMR on All-Cause Survival and Heart Failure Hospitalization After M-TEER

During a median follow-up of 49 (26-79) months, there were 91 deaths recorded. In addition, 67 patients were hospitalized at least once due to worsening heart failure symptoms.

**Figure 3:**
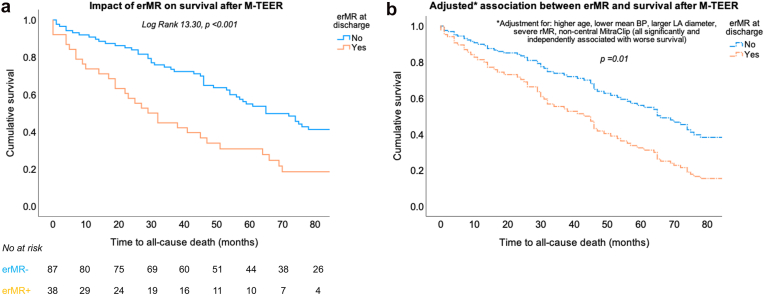
(a) Kaplan–Meier curves for time to all-cause death in patients with vs. without erMR at discharge after M-TEER. The value of the log rank test is given for the overall analysis with the respective *p* value. (b) Adjusted curves for time to all-cause death in patients with vs. without erMR at discharge after M-TEER. Adjustment was performed in multivariable Cox analysis, and the *p* value indicates the significance of the independent association between erMR and all-cause death. Abbreviations: BP, blood pressure; erMR, eccentric mitral regurgitation; LA, left atrial; MR, mitral regurgitation; M-TEER, transcatheter edge-to-edge mitral valve repair; rMR, residual mitral regurgitation.

**Figure 4:**
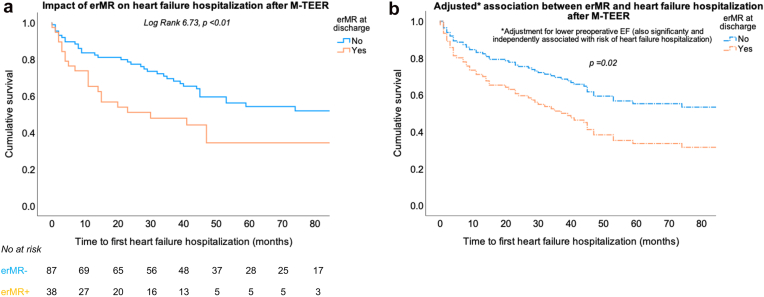
(a) Kaplan–Meier curves for the time to first heart failure hospitalization in patients with vs. without erMR at discharge after M-TEER. The value of the log rank test is given for the overall analysis with the respective *p* value. (b) Adjusted curves for the time to first heart failure hospitalization in patients with vs. without erMR at discharge after M-TEER. Adjustment was performed in multivariable Cox analysis, and the p value indicates the significance of the independent association between erMR and heart failure hospitalization. Abbreviations: EF, ejection fraction; erMR, eccentric mitral regurgitation; M-TEER, transcatheter edge-to-edge mitral valve repair.

In Kaplan–Meier analyses, patients with erMR early after M-TEER had a significantly higher risk of all-cause death (Figure 3a). They also had a higher probability of heart failure hospitalization compared to patients without erMR at discharge (Figure 4a). The median all-cause survival was 29 (18-40) months in the group with erMR at discharge vs. 65 (50-80) months in the group with no erMR (*p* < 0001). Survival was comparable in patients with erMR induced by M-TEER (32 [13-51] months) and patients with pre-existing erMR (27 [18-36] months) (*p* = 0.97). After the valve intervention, 63% of patients with erMR were hospitalized at least once with worsening heart failure vs. 49% of patients without erMR (*p* < 0.01). When the Kaplan–Meier analysis was run only in patients with mild residual regurgitation, erMR remained associated with a higher risk of death (log rank 4.74, *p* = 0.03). Moreover, when analyses were run separately in patients with initially ventricular functional MR (n = 68) vs. patients with either degenerative or atrial functional MR (n = 57), erMR at discharge predicted a worse long-term survival in both groups and a higher risk of worsening heart failure in patients with ventricular functional MR (all *p* < 0.05, Supplemental Figure).

In univariable Cox regression analyses, the following factors were significantly associated with worse all-cause survival after M-TEER: erMR, either severe rMR or moderate and greater rMR, lower mean BP, increased LV size, enlarged LA, absence of a central M-TEER device, and implantation of a medial device (Table 3). Neither the preoperative MR etiology nor the type of daily medication or the intraoperative or early postoperative mean mitral gradient was significantly associated with the long-term survival. In multivariable Cox analyses, an eccentric jet at discharge was associated with a 2-fold higher risk of all-cause death after adjustment for MR severity, age, mean BP, LV and LA size, known atrial fibrillation, and device position (Table 3, Figure 3b). Importantly, placement of a central device reduced the adjusted risk of death by 2.5-fold, whereas a medially placed device did not impact survival after adjustment for confounders. Moreover, moderate or greater rMR was no longer a predictor of survival after the jet eccentricity was considered, but severe rMR alone remained a strong negative predictive factor with an adjusted HR of 6.8 (95% CI 1.8-25.2), *p* < 0.01.

**Table 3:** Univariable and multivariable Cox regression analyses of factors associated with all-cause death after M-TEER

Variable	Univariable analyses		Multivariable analyses			
			Model 1		Model 2	
	HR (95% CI)	*p* value	HR (95% CI)	*p*	HR (95% CI)	*p* value
erMR at discharge	**2.19 (1.42-3.38)**	**<0.001**	**2.19 (1.28-3.74)**	**<0.01**	**2.01 (1.12-3.63)**	**0.02**
Age, years	**1.03 (1.00-1.06)**	**0.06**	**1.04 (1.00-1.08)**	**0.03**	**1.04 (1.01-1.08)**	**0.02**
Male sex	1.13 (0.71-1.78)	0.61				
Heart rate, bpm	1.01 (0.99-1.02)	0.59				
Mean BP, mmHg	**0.98 (0.97-1.00)**	**<0.01**	**0.98 (0.96-1.00)**	**0.04**	0.98 (0.96-1.00)	0.06
History of hypertension	0.85 (0.56-1.31)	0.47				
Atrial fibrillation	**1.48 (0.95-2.31)**	**0.09**	1.14 (0.62-2.11)	0.68	1.08 (0.57-2.02)	0.82
Diuretic	1.61 (0.83-3.11)	0.16				
ACE inhibitor or ARB	1.11 (0.63-1.97)	0.72				
Beta-blocker	1.71 (0.79-3.71)	0.18				
Etiology of preoperative MR: atrial vs. ventricular functional MR	0.85 (0.53-1.37)	0.51				
Etiology of preoperative MR: degenerative vs. ventricular functional MR	0.74 (0.41-1.34)	0.32				
LV end-systolic diameter, cm	**1.17 (1.00-1.37)**	**0.05**	1.13 (0.93-1.38)	0.23	1.08 (0.89-1.31)	0.47
LV EF, %	0.99 (0.98-1.01)	0.11				
LA maximum diameter, cm	**1.28 (1.06-1.55)**	**0.01**	**1.42 (1.11-1.82)**	**<0.01**	**1.41 (1.09-1.81)**	**<0.01**
Tricuspid maximum pressure gradient, mmHg	1.00 (0.99-1.02)	0.85				
Central device	**0.47 (0.25-0.88)**	**0.02**	**0.40 (0.20-0.79)**	**<0.01**		
Medial device	**1.98 (1.12-3.52)**	**0.02**			1.47 (0.72-2.98)	0.29
Number of devices (>1 device)	1.35 (0.94-1.93)	0.10				
Moderate or severe MR at discharge	**1.54 (1.02-2.34)**	**0.04**	0.97 (0.57-1.63)	0.90	1.09 (0.65-1.82)	0.75
Mean mitral gradient at discharge, mmHg	1.04 (0.91-1.19)	0.53				

*Notes. p* values below 0.1 in univariable analyses and below 0.05 in multivariable analyses are marked in bold text.

Abbreviations: ACE, angiotensin-converting enzyme; ARB, angiotensin receptor blocker; BP, blood pressure; CI, confidence interval; EF, ejection fraction; erMR, eccentric residual mitral regurgitation; HR, hazard ratio; LA, left atrial; LV, left ventricular; MR, mitral regurgitation; M-TEER, transcatheter edge-to-edge mitral valve repair.

In similar Cox analyses, erMR was associated with a 1.84 (95% CI 1.09-3.11, *p* = 0.02) higher risk of heart failure hospitalization after adjustment for LV EF (HR 0.9, 95% CI 0.97-1.00, *p* = 0.04), mean BP (HR 0.99, 95% CI 0.97-1.01, *p* = 0.17), and severe rMR (HR 0.52, 95% CI 0.07-3.89, *p* = 0.53) (Figure 4b). Other theoretically relevant preoperative and intraprocedural factors were not identified as predictors in univariable analyses and thus not further tested.

## Discussion

Based on data accumulated over a decade on patients who underwent M-TEER at our institution and had at least trace residual regurgitation at discharge, the current investigation shows that not only the quantity of the residual regurgitation, but also the jet morphology has a significant impact on patient outcomes. In our cohort, an erMR at discharge was present in 30% of patients and more commonly associated with medially placed clips and greater MR severity. However, erMR predicted a 2-fold higher risk of all-cause death over a median 4-year follow-up after adjustment for the severity of the residual jet and independently of other important predictors such as LA enlargement and the position of the implanted mitral devices. Our findings draw attention to the prognostic importance of intra-atrial flow dynamics after M-TEER and underscore the importance of precluding the formation of wall-hugging jets when implanting edge-to-edge valvular devices.

### Eccentric MR Jets in Native Valves and After M-TEER

Native mitral valves might present eccentric or wall-hugging jets due to degenerative disease with prolapse or asymmetric leaflet tethering in failed LVs (ventricular functional MR).^14^^,^^15^ They may also occur in cases of long-standing atrial functional MR characterized by displaced posterior mitral annulus and hamstrung posterior leaflet.^16^ These eccentric jets can be oriented along the atrial surface of the bottom mitral leaflet (leaflet-hugging jets) or so-called Coanda jets after the Romanian aerodynamicist Henri Coanda who first described how the tendency of a fluid jet to follow a nearby surface can be used in aircraft lift design.^17^ They can also be deflected jets, that is, jets that simply follow the direction of the flow determined by a prolapsed orifice.^18^ Coanda MR jets form due to the high flow turbulence and from regurgitant orifices with large width compared to height. Deflected jets form from regurgitant orifices with small width-to-height ratio and can cause large 3D vortices in the LA. Both types of eccentric MR are visualized hugging the atrial wall by color Doppler echocardiography, are challenging to quantify, and perturb the intra-atrial flow dynamics more than central jets.^18^^,^^19^

The changed mitral geometry following M-TEER might in selected cases create new or fail to repair existing regurgitant orifices that, depending on their width-to-height ratio and the jet turbulence, might result in Coanda erMR or deflected erMR jets. In our cohort, nearly half of the erMRs were induced by M-TEER. Moreover, placement of a MitraClip in the medial part of the valve was significantly associated with the presence of an erMR at discharge. This association was consistent regardless of the original MR etiology. This might be due to the increased challenges in achieving a perfect leaflet alignment in the medial part of the valve that is more chorda-rich than the central one, a possibility also supported by the finding that a centrally placed clip was prognostically beneficial. Moreover, in patients with erMR, MR was more often classified as more severe both pre-TEER (possibly explaining the lower BP values of these patients) and post-TEER. Both Coanda and deflected MR jets have been shown in an experimental setting to have a more severe impact on the LA flow dynamics at higher regurgitant volumes. This suggests that the combination of larger MR volume and eccentric jet morphology represents the most unfavorable post-M-TEER pattern.^18^ Interestingly, the degree of preoperative LA and LV remodeling was comparable between groups and therefore unlikely to have contributed per se to erMR.

### Eccentric rMR and Survival After M-TEER

The impact of eccentric MR on survival has scarcely been reported in native mitral valve disease and, to our knowledge, not explored in real-world data from patients treated by M-TEER. In atrial functional MR, patients with eccentric MR were found to have a higher risk of death, heart failure hospitalization, and mitral valve interventions after a short follow-up time of 0.7 years.^8^ In patients undergoing M-TEER, having more than mild rMR was demonstrated to be a negative prognostic factor.^20^^,^^21^ Our investigation shows, however, that not only the quantity of MR but also the quality of the rMR jet has an impact on survival, with presence of erMR early after M-TEER being associated with a doubling in the long-term adjusted risk of all-cause death. It was also associated with an 80% higher chance of hospitalization due to heart failure after M-TEER. This prognostic impact might theoretically be due to large 3D vortex rolls created by the eccentric jets in the LA as explained above. As such, perturbed LV flow dynamics with changed vortex strength has been related to adverse outcomes in patients with LV failure.^22^ However, the relation between eccentric MR jets and maladapted vortex dynamics in the LA has been scarcely studied and needs further exploration in computational and in vivo imaging studies of LA flow dynamics.

Importantly, the association between erMR and mortality held true after adjustment for MR severity and across the spectrum of MR etiologies. It was also significant after adjustment for higher age, lower BP, and left-heart remodeling. An increased preoperative LA size had a negative impact on long-term survival, adding to existing research on the importance of LA remodeling in patients referred to M-TEER.^23^^,^^24^ Notably, centrally placed clips were associated with 2.5-fold reduction in the adjusted risk of death, suggesting a lower risk of complications when addressing this area of the valve that includes a chorda-free central part of the anterior leaflet, and maybe also a long-term stabilizing effect.^25^

### Clinical Implications

Patients with significant degenerative or functional MR who are considered at high or prohibitive risk for surgical treatment may be suitable candidates for M-TEER. Ongoing studies are testing whether the indications for M-TEER might be extended to patients at moderate surgical risk.^5^ However, since at least trace rMR is a common finding after M-TEER, both the preprocedural and intraprocedural planning should focus on mitigating the long-term adverse impact of residual jets on clinical outcomes. Presently, 30% of patients undergoing M-TEER present with erMR and have reduced survival and increased risk of heart failure worsening after M-TEER. Our data support a more extensive use of preintervention simulations using preoperative 3D imaging data to better forecast the effect of percutaneous devices on mitral valve geometry. This type of planning should, among others, focus on avoiding the formation of regurgitant orifices through which Coanda or deflected erMR jets could form. Intraoperatively, increased attention should be dedicated to the possibility of leaflet misalignment when treating the medial part of the valve, and the device implantation strategy should be revised if eccentric jets form. Further research on the impact of preprocedural simulations on long-term clinical and imaging outcomes is warranted.

### Study Limitations

This study is a retrospective analysis of data from a single heart valve center and has the limitations inherent to this design. It reflects immediately the contemporary, real-world use of M-TEER and has the advantage of homogenous quality of data acquisition and analysis. We do not provide quantitative measures of MR severity as quantification by the proximal isovelocity surface area-method is not recommended following M-TEER and the volumetric assessment of MR volume by 2D echocardiography leads frequently to MR underestimation.^11^ However, the degree of MR was assessed using a multiparametric approach performed by experts in valvular heart disease management. As the exact cause of death could not be determined in all patients, we report associations with all-cause mortality.

## Conclusion

Early erMR is associated with reduced 4-year survival after M-TEER independent of the severity of residual regurgitation and after adjustment for the protective effect of centrally placed mitral valve devices. Increased awareness toward the negative prognostic impact of eccentric jets and careful preprocedural and intraprocedural planning might improve the long-term outcomes after M-TEER.

## CRediT Authorship Contributions

Barbara Rogge, Vegard Tuseth, Erik Packer, and Dana Cramariuc were involved in the conception and design. Barbara Rogge, Jon Herstad, Erik Packer, and Dana Cramariuc were involved in data acquisition. Knut Qvale Søndenaa, Ola Alexander Schei, Christian Engelsen Berg-Hansen, Rasmus Bach Sindre, and Dana Cramariuc were involved in analysis and interpretation of data. All coauthors have revised the manuscript critically and approved it for submission.

## Ethics Statement

The study was approved by the regional ethics committee (2019/35092) and was conducted in accordance with the revised Declaration of Helsinki. The need for informed consent was waived due to the retrospective nature of the investigation. The data that support the findings of this study are available from the corresponding author on reasonable request.

## Funding

This study has been partially funded by the 10.13039/501100007872Grieg Foundation. Dr Cramariuc receives clinical career research funds from the Norwegian Regional Health Authorities (project number F-13419).

## Disclosure Statement

Vegard Tuseth reports financial support was provided by the Grieg Foundation.

The other authors had no conflicts to declare.

## Figures and Tables

**Supplemental Figure 1:**
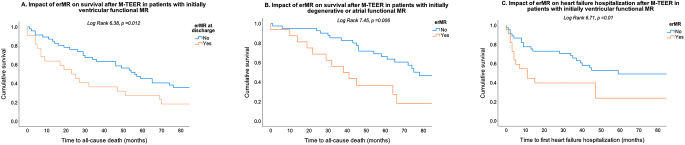
Kaplan–Meier curves for time to death (panels a and b) or time to first heart failure hospitalization (panel c) in patients with initially ventricular functional MR (panels a and c) or degenerative or atrial functional MR (panel b) and with vs. without erMR at discharge after M-TEER. The values of the log rank tests are given for the overall analysis with the respective *p* value. Abbreviations: erMR, eccentric mitral regurgitation; M-TEER, transcatheter edge-to-edge mitral valve repair.
